# Genetic analysis resolves differential diagnosis of a familial syndromic dilated cardiomyopathy: A new case of Alström syndrome

**DOI:** 10.1002/mgg3.1260

**Published:** 2020-05-12

**Authors:** Barbara Lombardo, Valeria D'Argenio, Emanuele Monda, Andrea Vitale, Martina Caiazza, Lucia Sacchetti, Lucio Pastore, Giuseppe Limongelli, Giulia Frisso, Cristina Mazzaccara

**Affiliations:** ^1^ Department of Molecular Medicine and Medical Biotechnology University of Naples “Federico II” Naples Italy; ^2^ CEINGE Advanced Biotechnologies Naples Italy; ^3^ Department of Translational Medical Sciences University of Campania ‘Luigi Vanvitelli’ Caserta Italy; ^4^ Cardiomyopathies and Heart Failure Department Monaldi Hospital University of Campania 'Luigi Vanvitelli' Naples Italy; ^5^ Department of Motor Science and Health University of Naples, Parthenope Naples Italy

**Keywords:** Alström syndrome, array CGH, idiopathic cardiomyopathy, mitochondrial, syndromic dilated cardiomyopathy, Usher syndrome, whole exome sequencing

## Abstract

**Background:**

Syndromic dilated cardiomyopathy (DCM) includes a group of complex disorders with a very heterogeneous genetic etiology, leading to delay in definitive diagnosis. Conversely, an early genetic diagnosis is very important in determining the disease course, the prognosis, and may guide personalized treatments and family counseling.

**Methods:**

We analyzed two brothers with a multisystemic disorder, including dilated cardiomyopathy, diabetes, bilateral neurosensorial hearing loss, and optic atrophy, using different genetic approaches, namely mitochondrial DNA sequencing, comparative genomic hybridization‐array (a‐CGH) and whole exome sequencing (WES).

**Results:**

Sequencing of the wide mitochondrial genome revealed, in both brothers, the known homoplasmic variant rs2853826 in the subunit 3 of the *NADH dehydrogenase* gene (*MT‐ND3*), whose pathogenicity was conflicting. Comparative genomic hybridization‐array analysis revealed in both patients and their father two heterozygous deletions in Phosphodiesterase 4d‐Interacting Protein (*PDE4DIP*) and Protocadherin‐related 15 (*PCDH15)* genes, respectively. The use of WES detected a pathogenetic mutation in *ALMS1*, enabling the definitive diagnosis of Alström syndrome.

**Conclusion:**

We demonstrated how the diagnosis of a complex heterogeneous disease may be difficult, due to several overlapping manifestations and the possible interaction of more genetic variants that could lead to a more severe and complex phenotype.

This paper strongly evidences how genomics is revolutionizing the diagnosis of rare complex disease, representing one of the most essential steps to enable a definitive diagnosis and to establish the etiology for diseases, such as syndromic DCM.

## Graphical abstract

The diagnosis of a complex heterogeneous disease may be difficult, due to several overlapping manifestations and the possible interaction of more genetic variants. By using different genetic approaches we obtained a definitive diagnosis of Alström syndrome in two brothers with a multisystemic disorder.

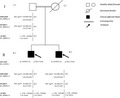

## 1 INTRODUCTION

Genetic diagnosis of inherited cardiomyopathy is particularly challenging, because of the exceeding genetic heterogeneity and the overlapping, variable and incompletely penetrant nature of the clinical presentations (Frisso et al., 2009; Girolami et al., 2018; Mazzaccara et al., 2018).

A rare form of syndromic dilated cardiomyopathy (DCM) is the Alström syndrome (AS) [OMIM 203800] (prevalence 1.4 cases per 1,000,000), a multisystem disorder, inherited as autosomal recessive trait, caused by mutations in the *ALMS1* (Zmyslowska et al., 2016) (OMIM 606,844). Typical Alström patient shows a characteristic age‐dependent emergence of symptoms, starting with nystagmus and cone‐rod dystrophy at birth, or in the first month of life, leading to blindness in infancy/childhood. Most patients (92%) show insulin resistance, usually developed between age 2 and 4 years, evolving into type 2 diabetes mellitus (T2DM) within a median age of 16 years (Marshall, Maffei, Collin, & Naggert, 2011). As many as 70% of Alström patients show sensorineural hearing loss in the first decade. Almost two thirds of AS patients show DCM: patients with infant onset of DCM and heart failure (48%) and patients developing DCM as adolescents or adults (17%) (Marshall et al., 2011). The very low prevalence and the age‐dependent phenotype generally delay AS diagnosis (Hoffman, Jacobson, Young, Marshall, & Kaplan, 2005). Furthermore, pleiotropic clinical features involve problems of differential diagnosis with other complex disorders, such as mitochondrial diseases (MDs), Bardet–Biedl syndrome, Leber congenital amaurosis, and Usher syndrome (Lenarduzzi et al., 2015).

In particular, the key clinical features usually observed in AS, which sensorineural hearing loss, T2DM, retinal dystrophy, and cardiomyopathy, are also the main manifestations related to mitochondrial disease, (Pineiro‐Gallego, Corton, Ayuso, Baiget, & Valverde, 2012) so that the “mitochondrial phenotype” is frequently overlapped with AS, making the AS diagnosis often delayed or misdiagnosed.

Based on clinical suspicion of mitochondrial cardiomyopathy, we sequenced the whole mitochondrial genome (mtDNA) of two brothers with multisystemic disorder which clinical findings were strongly suggestive of mitochondrial disorder. Furthermore, as chromosomal rearrangements may be causative of the “mitochondrial phenotype,” by disrupting one or more genes involved in mitochondrial government (Niyazov, Kahler, & Frye, 2016), we have subsequently investigated the presence of nuclear macro‐deletions, by array CGH, in the patients and their father. However, the use of whole exome sequencing (WES) enabled the definitive diagnosis of Alström syndrome and established the etiology of the complex phenotype.

The aim of this report is to suggest the whole exome analysis to resolves differential diagnosis of familial syndromic DCM and to uncovers potential confounding disorders.

## 2 CLINICAL PRESENTATION

We report two male brothers (GA and GL), both showing a complex multisystemic phenotype. Particularly, GA was suffering from retinitis pigmentosa, leading to blindness at 14 years, and from bilateral neurosensorial hearing loss, since he was 27 years. Based on these findings, clinical diagnosis of Usher Syndrome (USH) was suspected. No molecular analysis was conducted at that time. At 35 years of age, a cardiological evaluation showed the presence of atrial fibrillation and dilated cardiomyopathy. In the same period, diagnosis of type II diabetes and hypothyroidism was made. At 47 years, he experienced an ischemic stroke with facial–brachial–femoral left transient hemiparesis and multiple right renal infarcts. Two years later he underwent internal cardioverter defibrillator (ICD) implantation.

The brother GL showed retinitis pigmentosa since birth, resulting in blindness at 20 years, and bilateral neurosensorial hearing loss. Moreover, similar to GA, he showed the presence of atrial fibrillation, dilated cardiomyopathy and diabetes since he was 30 years old.

**Figure 1:**
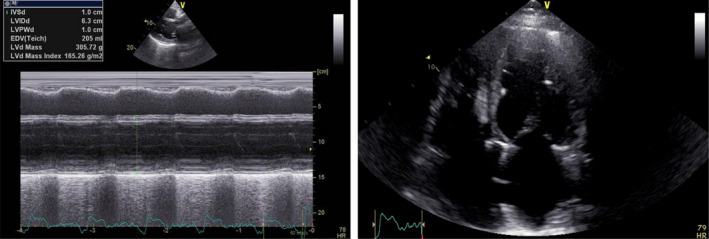
Transthoracic echocardiogram from GA patient. Transthoracic echocardiogram from parasternal long‐axis view (M‐mode) (on the left) and from apical four‐chamber view (on the right) showing a severe dilation of the left ventricle (63 mm) and of the left atrium

The patients’ mother was apparently healthy until the age of 30, when she died as a consequence of a hepatic carcinoma. Their father (GG), 86 years old, did not suffer from pathologies. At 57 (GA) and 55 (GL) years old, the brothers were recruited at the Inherited and Rare Cardiovascular Disease Clinic, University of Campania Luigi Vanvitelli, Naples, (Italy) for the etiological framing. Both brothers showed a severe dilated cardiomyopathy (Figure 1); conversely no evidence of clinically silent dilated cardiomyopathy was observed in their father. Based on the presence of multiple organ dysfunction, a mitochondrial cardiomyopathy was suspected, often being accompanied by concurrent diseases, including diabetes, bilateral neurosensorial hearing loss, and retinitis pigmentosa (Lenarduzzi et al., 2015; Limongelli, Masarone, & Pacileo, 2017).

The family's members were then referred to the CEINGE–Advanced Biotechnology/Department of Molecular Medicine and Medical Biotechnology, University of Naples Federico II (Italy) for the genetic investigation.

## 3 GENETIC ANALYSIS

We extracted genomic DNAs from both peripheral blood samples and buccal cells of the two brothers, by using the Kit Nucleon BACC 2 (Illustra DNA Extraction Kit BACC2‐GE Healthcare). Similarly, we obtained genomic DNA from the peripheral blood sample of the father. Written informed consent to perform genetic analysis was obtained from all subjects, according to the second Helsinki Declaration (Leggiero et al., 2013).

### 3.1 Mitochondrial DNA sequencing

The whole mitochondrial genome from the two brothers was amplified by long‐PCR and subsequently sequenced by Sanger method, as previously reported (Mazzaccara et al., 2012). The achieved sequences were analyzed using Codon Code program (Codon Code Aligner vs. 5.1.5) to compare mtDNA sequences from the swab and blood samples, first to the revised Mitochondrial Cambridge Reference Sequence (rCRS NC_012920) and then to 70 control subjects, present in our database (Mazzaccara et al., 2012).

**Figure 2:**
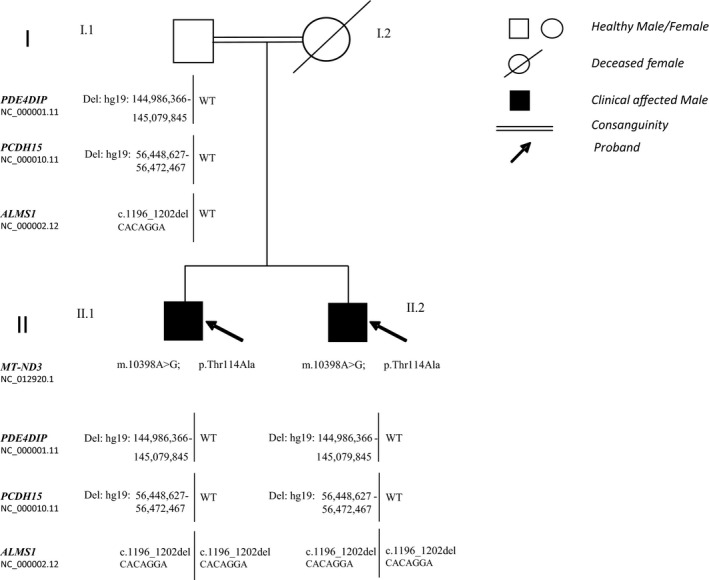
Familial pedigree. *MT‐ND3*, PDE4DIP, *PCDH15*, *ALMS1* mutations pattern of the probands and their father and clinical phenotype. II.1: retinitis pigmentosa, bilateral neurosensorial hearing loss, dilated cardiomyopathy, diabetes, hypothyroidism. II.2: retinitis pigmentosa, bilateral neurosensorial hearing loss, dilated cardiomyopathy and diabetes. MT‐ND3: NADH dehydrogenase gene, subunit 3, NC_012920.1; PDE4DIP: Phosphodiesterase 4d‐Interacting Protein gene, NC_000001.11; PCDH15: Protocadherin‐related 15 gene, NC_000010.11; ALMS1: Alström gene, NC_000002.12

The sequences revealed the presence of the known homoplasmic variant m.10398A>G [c.340A>G; p.Thr114Ala; rs2853826] in the subunit 3 of the *NADH dehydrogenase* gene (*MT‐ND3*; OMIM 516,002, Gene ID: 4,537, NC_012920.1) (Figure 2), reported in the MITOMAP (http://www.mitomap.org) and 1,000 genome (http://www.internationalgenome.org/1000‐genomes‐browsers) databases.

The m.10398A>G frequency varies significantly among different populations, ranging from 10% to 20% in Caucasians (Pezzotti et al., 2009). In our previously analyzed 70 control subjects, we found this substitution with Minor Allele Frequency (MAF): 17%, similar to frequency of the Caucasian population. The rs2853826 is described in correlation to several diseases, including type II diabetes and metabolic syndrome, but with conflicting results regarding both the correlation between the polymorphism and the disease risk, and the possible association with other genetic variants that all together may contribute to make this variant pathogenic (Juo et al., 2010).

### 3.2 Array comparative genomic hybridization (a‐CGH)

Because of the difficulty to establish, in our patients, a mitochondrial genotype–phenotype correlation, a high‐resolution comparative genomic hybridization‐array (a‐CGH) analysis was performed, to identify large‐scale rearrangements (Di Stefano et al., 2015; Iossa et al., 2015; Zebisch et al., 2015). Sometimes, copy‐number variants (CNVs) may be identified in patients suffering from DCM with other multiple anomalies (Norton et al., 2011). Genomic DNAs, from both brothers and their healthy father, were analyzed with the CGH 1×1M Microarray (Agilent Technologies). DNA digestion, labeling and hybridization were performed according to the manufacturer's protocols.

In both brothers we detected a heterozygous deletion in the region 1q21.1 (hg19:144,986,366–145,079,845) of approximately 93,5 Kb covering the Phosphodiesterase 4d‐Interacting Protein (*PDE4DIP*) gene (OMIM 608,117, Gene ID: 9,659, NC_000001.11) encoding for the protein PDE4DIP (also called cardiomyopathy‐associated protein 2 or more commonly myomegalin), serving to anchor phosphodiesterase 4D (PDE4D) to the Golgi/centrosome region of the cells(Verde et al., 2001). The myomegalin is associated with both increased risk for ischemic stroke and Usher syndrome.

Interestingly, we found in both patients another heterozygous deletion in the region 10q21.1 (hg19:56,448,627–56,472,467), of approximately 23.8 Kb which involves protocadherin‐related 15 gene (*PCDH15*) (OMIM 605,514, Gene ID: 65,217, NC_000010.11), coding *PCDH15*, a member of the cadherin superfamily, which plays an essential role in maintenance of normal retinal and cochlear function (Figure 2). Homozygous or double heterozygous mutations in this gene result in hearing loss and Usher Syndrome. However, the presence of these alterations also in the healthy father excludes the diagnosis of Usher Syndrome due to digenic inheritance (Ahmed et al., 2008; Schrauwen et al., 2018). Collectively, the results of the whole mtDNA and large‐scale DNA investigations failed to obtain known causes, explaining the heterogeneous complex disorder. At that time, NGS methodology has been available at our institute, therefore we recalled patients for a diagnostic deepening. On this occasion they referred a remote relationship among the parents.

### 3.3 Whole exome sequencing

gDNA samples, from both brothers and their father, were used to prepare univocally tagged exome enriched libraries, according to manufacturer's instructions (Agilent technologies). The sequencing run was carried out on the NextSeq500 System using the High Output PE 300 Cycles flow‐cell (Illumina). The obtained FASTQ files were analyzed using the Alissa software (Agilent Technologies, Inc. 2019 ‐ v1.0.2–10).

An average of 114,046,339 reads was obtained for each of the three family members (GG 115,214,281 reads, GL 116,487,552 reads, and GA 110,437,184 reads) and more than 96% of these reads passed all the quality filters. The average read depth in the analyzable target regions was 150× for all patients and the percentage of analyzable target regions covered by at least 20 reads was up to 96%. The number of DNA variants found was about 85,000 per sample (86,173 for GG, 85,530 for GL, and 84,853 for GA); 99.5% for all passed quality checks.

The analysis pipeline resulted in a short list of seven possible pathogenic variants (Table S1), among which the variant in the *ALMS1* (OMIM: 606,844, Gene ID: 7,840, NC_000002.12) NM_015120.4: c.1196_1202delCACAGGA; ENST00000613296.5; NP_055935.4: p.Thr399LysfsTer11 (rs761292021, MAF: 0.00001 in GnomAD exome). It was the only compatible with the patient's pleiotropic phenotype and the inheritance matters, being homozygous in both patients and heterozygous in their unaffected father (Figure 2).

## 4 DISCUSSION

Herein, we describe two brothers with a rare complex disease, whose multiple symptoms arose over a time from birth to adulthood age. The combined deaf‐blindness and the later onset of DCM and diabetes suggested a mitochondrial disease, as a start. For this apparently “mitochondrial phenotype”, we considered appropriate to investigate the mitochondrial genome alterations and the nuclear rearrangements. However, both whole mtDNA sequencing and a‐CGH failed to identify a mutation associated with the clinical features. At a later time, WES was performed, showing the homozygous pathogenic mutation c.1196_1202delCACAGGA, in *ALMS1* in both brothers, one copy inherited from their heterozygous father. To our current knowledge, based on literature data research, variations database consultation, (i.e., HGMD professional mutation database, Exome Variant Server Database) there are not references or clinical information about the variant, however, it is present in National Center for Biotechnology Information, https://www.ncbi.nlm.nih.gov/ (accessed February 2020) and classified as pathogenic, according to the American College of Medical Genetics (ACMG) criteria (Richards et al., 2015). Actually, *ALMS1* is the only gene associated with the Alström syndrome and pathogenic variants are usually nonsense or frameshift mutations, resulting in a truncated protein (Marshall et al., 2015). According to the latter, the mutation found in our patients is the deletion of seven nucleotides, that is, CACAGGA, in the exon 5 of 23 of *ALMS1*; as a consequence, it causes a frameshift at protein level and a premature stop at codon 410 of ALMS1 protein, the wild type being of 4,168 amino acids. Thus, its pathogenic effect seems to be strongly supported.

This result allowed establishing the definitive molecular diagnosis of Alström syndrome, which was also in agreement with the reported consanguinity.

*ALMS1* gene encodes ALMS1, a large ubiquitous protein, which function is not widely known; it seems to be implicated in intracellular trafficking and ciliary function, and seems also to be involved in nonciliary functions, including actin organization and endosomal trafficking. ALMS1 is also needed for trafficking of the insulin receptor glucose transporter type 4 (GLUT4) to the plasma membrane, adipogenesis, and regulation of pancreatic beta cell mass. Its absence causes diabetes, a common feature of Alström syndrome (Hearn, 2019). Furthermore, deficiency of ALMS1 in childhood impairs postnatal cardiomyocyte cell cycle arrest, activating Wnt/β‐catenin cascade and inducing genes transcription that promote cell cycle proliferation (Brofferio et al., 2017; Shenje et al., 2014). However, concurrent metabolic alterations may contribute to progression of cardiac disease. Early diagnosis of AS is arduous in youth, due to the overtime onset of the many clinical features; moreover, the development of DCM in adulthood, as in our patients, may contribute to the delayed correct diagnosis or misdiagnosis.

Genotype–phenotype correlation is complex to establish in AS, due to modifying genes, inherited independently from the *ALMS1*, as has been reported in other overlapping phenotypes (Marshall et al., 2015; Schrauwen et al., 2018). In this context, the possible additive effects of the two digenic heterozygous deletions in *PDE4DIP* and *PCD15*, detected in both brothers, should be considered. Indeed, rare cases of Usher patients carry heterozygous pathogenic variants in two or three different genes, including *PCD15* or *PDE4DIP* (Schrauwen et al., 2018). Furthermore, the rs2853826 polymorphism in the mtDNA is described in correlation with several diseases, including T2DM and metabolic syndrome; thus we cannot exclude that, in the genetic context of our patients, this variant could play a worsening role in the phenotype (Juo et al., 2010).

In the era of the personalized medicine, WES allows a genotype first approach and often represents the more cost effective and efficient tool to reach a definitive diagnosis of syndromic diseases. WES also may undoubtedly avoid delayed diagnosis and allow a better follow‐up and presymptomatic interventions. In our context, in addition to the *ALMS1* mutation driving the phenotype, a plurality of variants in different genes (*MT‐ND3, PDE4DIP,* and *PCD15*) may determine more severe and complex manifestations (Biagini et al., 2014). In conclusion, the clinical and genetic work‐up on our patients showed how the diagnosis of a complex heterogeneous disease may be difficult, due to several overlapping manifestations and the possible interaction of more genetic variants. The paper strongly evidences how genomics is revolutionizing the diagnosis of rare complex disease. In the era of a huge claim for personalized medicine, comprehensive genetic studies represent one of the most representative steps to enable a definitive diagnosis and to establish the etiology for syndromic disease such as syndromic dilated cardiomyopathy.

## CONFLICT OF INTEREST

The authors declare no conflict of interest.

## AUTHOR CONTRIBUTIONS

GF, CM, BL, and VD wrote the manuscript and approved the final version. EM, LS, LP, and GL critically revisited the manuscript for important intellectual content. MC and AV involved in literature, laboratory and clinical data collection. All authors critically reviewed the manuscript.

## Supporting information

TableS1

## Data Availability

The authors confirm that the data supporting the findings of this study are available within the article and/or its supplementary material. Additional data, scripts, and other products used to generate the analyses presented in the paper are available from the corresponding authors (CM and GF) on request.
