# Supplementary material for: Genetic analysis resolves differential diagnosis of a familial syndromic dilated cardiomyopathy: A new case of Alström syndrome
Source: Mol Genet Genomic Med. 2020 May 12;8(7):e1260. doi: 10.1002/mgg3.1260 (PMC7336746; doi:10.1002/mgg3.1260)
Supplement: Supplementary file 1 — TableS1 [file MGG3-8-e1260-s001.doc]

**Supplemental Table 1.** List of pathogenetic/likely pathogenetic variants found in the three subjects (GA and GL: brothers; GL: father). In red the mutation found in *ALMS1* gene, associated with Alström syndrome.

| **Chr** | **Gene** | **cDNA** | **Protein** | **dbSNP** | **Exon** | **Classification*** | **Associated phenotype** | **GG** | **GA** | **GL** |
| --- | --- | --- | --- | --- | --- | --- | --- | --- | --- | --- |
| 1 | ***PCSK9*** | c.137G>T | p.R46L | rs11591147 | 1 | Pathogenic | Hypocholesterolemia | HET | HET | HET |
| 2 | ***ALMS1*** | c.1196_1202del CACAGGA | p.T400Kfs | rs761292021 | 5 | **Likely pathogenic** | **Alström syndrome** | **HET** | **HOM** | **HOM** |
| 3 | ***IQCB1*** | c.1178T>A | p.I393N | rs1141528 | 12 | Likely pathogenic | Nephronophthisis | HET | HET | HET |
| 4 | ***KLKB1*** | c.428G>A | p.S143N | rs3733402 | 5 | Pathogenic | Autoimmunity, Prolonged partial thromboplastin time | HOM | HOM | HOM |
| 6 | ***CCDC170*** | c.1810G>A | p.V604I | rs6929137 | 10 | Likely pathogenic​ | Estrogen resistance | HOM | HOM | HOM |
| 14 | ***GALC*** | c.1162-4delT | -------------- | rs11300320 | 11 | Likely pathogenic | Abnormality of brain morphology | HOM | HOM | HOM |
| 16 | ***ABCC6*** | c.451A>G | p.R151G | rs72657698 | 7 | Likely pathogenic | Pseudoxantoma | WT | HET | HET |

*based on ClinVar evaluation; HET: heterozygous; HOM: homozygous. GA, GL, Brothers; GG, Father
